# Effects of Wutou Decoction on DNA Methylation and Histone Modifications in Rats with Collagen-Induced Arthritis

**DOI:** 10.1155/2016/5836879

**Published:** 2016-03-06

**Authors:** Ya-Fei Liu, Cai-Yu-Zhu Wen, Zhe Chen, Yu Wang, Ying Huang, Yong-Hong Hu, Sheng-Hao Tu

**Affiliations:** ^1^Institute of Integrated Traditional Chinese and Western Medicine, Tongji Hospital, Tongji Medical College, Huazhong University of Science and Technology, 1095 Jiefang Avenue, Wuhan, Hubei 430030, China; ^2^Department of Nephrology, The First Affiliated Hospital of Zhengzhou University, 1 Jianshe East Road, Zhengzhou, Henan 450052, China; ^3^Hubei University of Chinese Medicine, 1 Huangjiahu West Road, Wuhan, Hubei 430065, China

## Abstract

*Background*. Wutou decoction (WTD) has been wildly applied in the treatment of rheumatoid arthritis and experimental arthritis in rats for many years. Epigenetic deregulation is associated with the aetiology of rheumatoid arthritis; however, the effects of WTD on epigenetic changes are unclear. This study is set to explore the effects of WTD on DNA methylation and histone modifications in rats with collagen-induced arthritis (CIA).* Methods*. The CIA model was established by the stimulation of collagen and adjuvant. The knee synovium was stained with hematoxylin and eosin. The DNA methyltransferase 1 (DNMT1) and methylated CpG binding domain 2 (MBD2) expression of peripheral blood mononuclear cells (PBMCs) were determined by Real-Time PCR. The global DNA histone H3-K4/H3-K27 methylation and total histones H3 and H4 acetylation of PBMCs were detected.* Results*. Our data demonstrated that the DNMT1 mRNA expression was significantly lowered in group WTD compared to that in group CIA (*P* < 0.05). The DNA methylation level was significantly reduced in group WTD compared to that in group CIA (*P* < 0.05). Moreover, H3 acetylation of PBMCs was overexpressed in WTD compared with CIA (*P* < 0.05).* Conclusions*. WTD may modulate DNA methylation and histone modifications, functioning as anti-inflammatory potential.

## 1. Introduction

Rheumatoid arthritis (RA) is a systemic autoimmune disease of unknown aetiology which is characterized by swelling, pain, stiffness, and deformity of peripheral joints [1]. Environmental factors and epigenetic deregulation are associated with the etiopathology of RA [2]. Epigenetics is defined as stable and heritable changes in gene expression which occur without a change in DNA sequence [3]. The predominant epigenetic mechanisms are DNA methylation, histone modification, and chromatin remodeling. It has been extensively demonstrated that epigenetics plays an important role in the pathogenesis of RA [4–8].

Moreover, various TCM-based herbal formulas and the extracts, such as Wutou decoction (WTD) [9], Xinfeng capsule [10], and* Tripterygium wilfordii* Hook F [11], have been employed in ameliorating articular and extra-articular manifestations of RA. WTD is constituted of six individual herbs which are prepared as seen in Table 1. Clinical studies demonstrated that WTD which was described in a famous TCM monograph Synopsis of Prescriptions of the Golden Chamber in Han Dynasty of China has been wildly applied for the treatment of RA [12], sciatica [13], and scapulohumeral periarthritis [14]. Meanwhile,* in vivo* animal experiments also validated that WTD or its derivatives significantly alleviate swelling, arthritis index, and hyperaemia in rats with adjuvant-induced arthritis [15–18].

In traditional Chinese medicine (TCM), RA falls into the category of arthromyodynia caused by wind, cold, and dampness which was described in the Yellow Emperor's Classic of Internal Medicine. WTD is capable of dispelling dampness by warming the channels and alleviating pain by dispersing coldness which is employed in the treatment of RA. And yet, the impacts of WTD on epigenetic changes are unknown. The study aims to explore the effect of WTD on DNA methylation and histone modifications in rats of collagen-induced arthritis (CIA).

## 2. Materials and Methods

### 2.1. Reagents and Main Devices

Bovine type II collagen (catalog: 20022) and incomplete Freund's adjuvant (catalog: 7002) were purchased from Amyjet Scientific Inc., China. The components of WTD except* Herbal Ephedrae* (Chinese Herbal Powder, Beijing Tcmages Pharmaceutical Co., Ltd., China) and* Herbal Ephedrae* were provided by Tongji Hospital. TRIzol Reagent (Invitrogen, 15596026), DNase I (Fermentas, EN0521), RevertAid Reverse Transcriptase (Fermentas, EP0442), dNTP (Fermentas, R0191), RiboLock RNase Inhibitor (Fermentas, E00381), All-in-One*™* qPCR Master Mix (GeneCopoeiaTM, AOPR-1200), DNA Extraction Kit (Aidlab Biotechnologies Co., Ltd., China, catalog: DN07), and Lymphocyte Separation Medium (Tian Jin Hao Yang Biological Manufacture Co., Ltd.) were ordered from Wuhan Boster Company, China. MethylFlash*™* Methylated DNA Quantification Kit (catalog: P-1034), EpiQuik*™* Total Histone Extraction Kit (catalog: OP-0006), EpiQuik*™* Global Pan-Methyl Histone H3-K4 Quantification Kit (catalog: P-3028), EpiQuik*™* Global Pan-Methyl Histone H3-K27 Quantification Kit (catalog: P-3044), EpiQuik*™* Total Histone H3 Acetylation Detection Fast Kit (catalog: P-4030), and EpiQuik*™* Total Histone H4 Acetylation Detection Fast Kit (catalog: P-4032) were purchased from Amyjet Scientific Inc., China.

Electric Homogenizer (Glas-Col, USA); Ice Machine (AF-100AS, Scotsman, USA); Centrifuge (2-16K, Sigma, USA); Nucleic Acid/Protein Analyzer (DU730, Beckman Coulter, Inc., California, USA); Microplate Reader (BioTek Synergy2, Vermont, USA); Heated Circulating Bath (DKB-600B, Keelrein instrument Co., Ltd., China); MiliporeRiOs 5 Water Purification Systems (Millipore, USA); Applied Biosystems StepOne Real-Time PCR System (Applied Biosystems, California, USA); and Esco Airstream Class II Biological Safety Cabinet (Beijing, China) were used.

### 2.2. Animals and Rearing Conditions

Five-week-old, weighted 130–150 g female Wistar rats (*n* = 45), SPF grade, were provided by the Center for Disease Control and Prevention of Hubei province (the animal certificate SCXK no. 2008-0005) and fed in the barrier system according to The Guidelines for the Care and Use of Animals in Research enforced by Hubei Municipal Science and Technology Commission. All protocols were approved by the Institutional Animal Care and Ethics Committee of Tongji Medical College, Huazhong University of Science and Technology. Food and water were given* ad libitum* throughout the experiment. The rats were caged in a standard barrier system with a 12 h light/dark cycle.

### 2.3. Grouping and Treatment

After 7 days of acclimation, the animals were randomly divided into three groups: normal group (N), CIA group, and WTD group (4.2 g/kg/d) with 15 rats in each group. The rat doses of treatment group were converted from human doses (Chinese Pharmacopeia, 2010) based on body surface areas. The final concentration of treatment group was 0.42 g/mL for WTD group. Oral gavage was performed once a day from day 28 after the initial immunization for four weeks. Rats in groups N and CIA were orally administered with the same volume of distilled water. The rats were fasted for 12 h but permitted water* ad libitum* before blood collection.

### 2.4. CIA Model Establishment and Arthritis Index Assessment

CIA model was established according to the manufacturer's standard protocols and previous research [19]. In addition to group N, CIA model was established in the other two groups. Bovine type II collagen was emulsified in an equal volume of incomplete Freund adjuvant, and 0.2 mL of the emulsion was injected subcutaneously about 3 cm distal from the base of the tail to induce arthritis. To ensure a high CIA incidence, a booster immunization (administer 0.1 mL of the emulsion subcutaneously in the tail) was performed two weeks after the initial immunization about 1.5 cm distal from the base of the tail.

**Figure 1:**
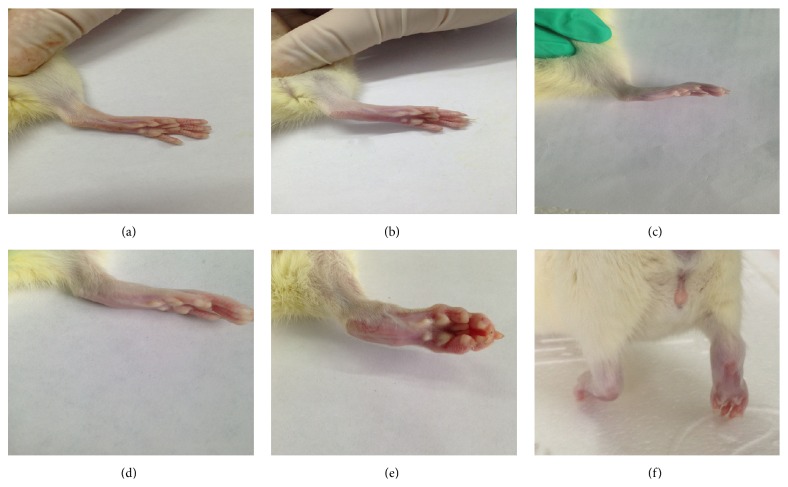
Arthritis index assessment: (a) 0; (b) 1; (c) 2; (d) 3; (e) 4; (f) right hind paw which is unable to bear weight.

As shown in Figure 1, the arthritis severity was evaluated by applying a previous published scoring system [20]. Once arthritis occurred, the arthritis index (AI) was evaluated twice a week for arthritis development. The arthritis score for each rat was the sum of the scores for two hind paws.

### 2.5. Sampling

At sixty minutes after the last intragastric administration, the rats were narcotized with 10% chloral hydrate by intraperitoneal injection. Blood was extracted from aorta abdominalis. The synovium isolated from knee joints was fixed in 4% paraformaldehyde for 12 h and embedded in paraffin. Paraffin-embedded synovial tissues were cut in 5 *μ*m thick sections and stained with hematoxylin and eosin. The sections were evaluated by two independent pathologists. The peripheral blood mononuclear cells (PBMCs) were separated by Lymphocyte Separation Medium using density gradient centrifugation method.

### 2.6. Real-Time PCR for PBMCs DNA Methyltransferase 1 (DNMT1) and Methylated CpG Binding Domain 2 (MBD2) Expression

Total RNA was extracted from PBMCs with TRIzol Reagent consistent with the manufacturer's instructions. RNA purity and concentration were measured by 1.8% agarose gel electrophoresis and Nucleic Acid/Protein Analyzer. cDNA was synthesized in accordance with the manufacturer's instructions. The cDNA was stored at −20°C prior to PCR amplification. Real-Time PCR reactions were performed applying StepOne Real-Time PCR System by following the manufacturer's instructions. Thermal cycler protocol: stage 1, Reps 1 95°C 10 min; stage 2, Reps 40 95°C 10 s, 60°C 20 s, and 72°C 20 s. The data were analyzed by employing 2^−ΔΔct^ method. The primers were designed in accordance with published sequences (Table 2).

### 2.7. Determination of Global DNA Methylation of PBMCs

Total DNA was extracted from PBMCs with DNA Extraction Kit consistent with the manufacturer's instructions. DNA purity and concentration were measured by 1% agarose gel electrophoresis and Nucleic Acid/Protein Analyzer. The global DNA methylation of PBMCs was detected according to the manufacturer's instructions. The amount and percentage of methylated DNA in total DNA were calculated using the following formulas: 5-mC (ng) = (Sample OD − ME3 OD)/(Slope × 2) or 5-mC% = (5-mC Amount (ng)/S) × 100%.

### 2.8. Determination of Global Pan-Methyl Histones H3-K4 and H3-K27 of PBMCs

Total histones were extracted from PBMCs with Histone Extraction Kit according to the manufacturer's instructions. Histone concentration was measured by BCA protein assay kit. The global histones H3-K4 and H3-K27 methylation of PBMCs was detected according to the manufacturer's instructions. The amount and percentage of mono-, di-, and trimethylated H3-K4 and H3-K27 were calculated applying the following formulas: Methylation% = OD (treated (tested) sample − blank)/OD (untreated (control) sample − blank) × 100% or Amount (ng/mg protein) = (OD (sample − blank)/Protein (*μ*g) × slope) × 1000.

### 2.9. Determination of Total Histones H3 and H4 Acetylation of PBMCs

Total histones were extracted from PBMCs with histone extraction kit in accordance with the manufacturer's instructions. Histone concentration was measured by BCA protein assay kit. The total histones H3 and H4 acetylation of PBMCs was detected according to the manufacturer's instructions. The amount and percentage of acetyls H3 and H4 were calculated using the following formulas: Acetylation% = OD (treated (tested) sample − blank)/OD (untreated (control) sample − blank) × 100% or Amount (ng/mg protein) = (OD (sample − blank)/Protein (*μ*g) × slope) × 1000.

### 2.10. Statistical Analysis

All data with a normal distribution were presented as mean ± standard deviation and analyzed with aid of SPSS17.0 statistical software. Statistical significance was determined by one-way analysis of variance (ANOVA). For data with equal variances assumed, ANOVA followed by LSD test was applied. For data with equal variances not assumed, ANOVA followed by Dunnett's *T*3 test was adopted. A probability of less than 0.05 was considered to be statistically significant.

## 3. Results

### 3.1. The Hematoxylin and Eosin Staining of Synovium Corresponding to AI Score

**Figure 2:**
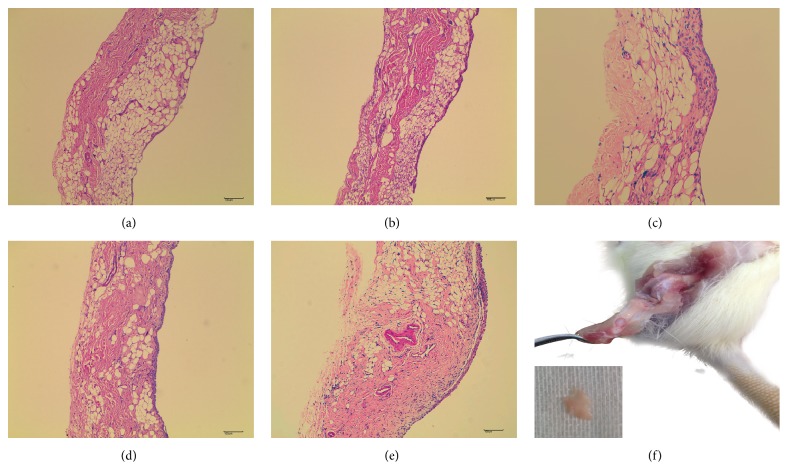
The hematoxylin and eosin staining of synovium corresponding to arthritis index score. (a), (b), (c), (d), and (e) represent the arthritis index scores 0, 1, 2, 3, and 4, respectively. (f) The process of isolating knee synovium.

The histopathological characteristics of synovial membrane corresponding to AI score were presented as follows: 0 (AI score): no alteration; 1 (AI score): the synovial lining cells form 2-3 layers; 2 (AI score): the synovial lining cells form 4-5 layers (multinucleated cells might occur); 3 (AI score): the synovial lining cells form 5–8 layers, hyperplastic synovial stroma, and pannus formation; 4 (AI score): the synovial lining cells form more than 8 layers, numerous inflammatory cell infiltration, and neovascularization (Figure 2).

### 3.2. Expression of PBMCs DNMT1 and MBD2 mRNA

**Figure 3:**
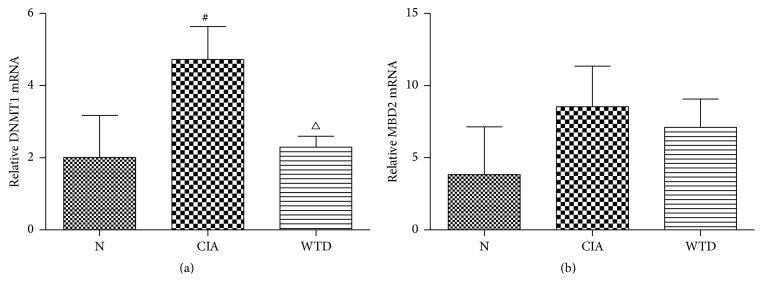
(a) Expression of PBMCs DNMT1 mRNA and (b) expression of PBMCs MBD2 mRNA. Values are mean ± SD. ^#^ *P* < 0.05 compared with group N and ^△^ *P* < 0.05 compared with group CIA. N: normal; CIA: collagen-induced arthritis; WTD: Wutou decoction; PBMCs: peripheral blood mononuclear cells; DNMT1: DNA methyltransferase 1; MBD2: methyl CpG binding domain 2.

Compared with group N, the DNMT1 mRNA expression was significantly elevated in group CIA (*P* < 0.05). The DNMT1 mRNA expression was significantly lowered in group WTD compared to that in group CIA (*P* < 0.05) (Figure 3(a)). Compared with group N, the MBD2 mRNA expression was increasing in group CIA; however, there was no significant difference between the two groups. Compared with group CIA, there was a reduction in group WTD; however, there was no significant difference between the two groups (Figure 3(b)).

### 3.3. Expression of Global DNA Methylation Level in PBMCs

**Figure 4:**
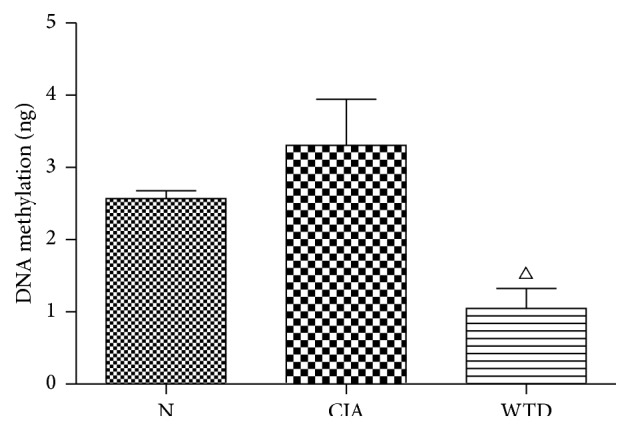
Expression of global DNA methylation level in PBMCs. Values are mean ± SD. ^△^ *P* < 0.05 compared with group CIA. N: normal; CIA: collagen-induced arthritis; WTD: Wutou decoction; PBMCs: peripheral blood mononuclear cells.

Compared with group N, the global DNA methylation level was enhancing in group CIA; however, there was no significant difference between the two groups. Compared with group CIA, the global DNA methylation level was significantly reduced in group WTD (*P* < 0.05) (Figure 4).

### 3.4. Expression of Global Pan-Methyl Histones H3-K4 and H3-K27 in PBMCs

**Figure 5:**
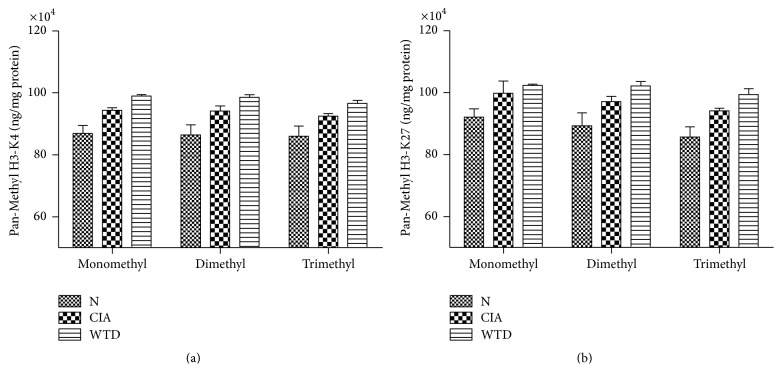
Expression of Global Pan-Methyl Histones H3-K4 and H3-K27 in PBMCs. Values are mean ± SD. N: normal; CIA: collagen-induced arthritis; WTD: Wutou decoction; PBMCs: peripheral blood mononuclear cells.

Compared with group N, the mono-, di-, and trimethylated H3-K4 and H3-K27 were increasing in group CIA; however, there was no significant difference between the two groups. Compared with group CIA, the mono-, di-, and trimethylated H3-K4 and H3-K27 were enhancing in group WTD; however, there was no significant difference between the two groups (Figures 5(a) and 5(b)).

### 3.5. Expression of Total Histones H3 and H4 Acetylation in PBMCs

**Figure 6:**
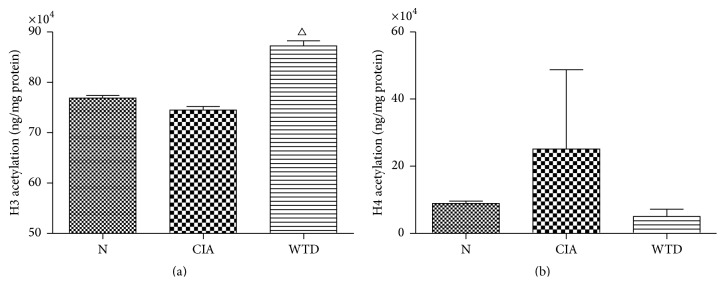
Expression of total histones H3 and H4 acetylation in PBMCs. Values are mean ± SD. ^△^ *P* < 0.05 compared with group CIA. N: normal; CIA: collagen-induced arthritis; WTD: Wutou decoction; PBMCs: peripheral blood mononuclear cells.

Compared with group N, the total histone H3 acetylation in group CIA was no significant alteration. The total histone H3 acetylation in group WTD was significantly elevated compared to that in group CIA (*P* < 0.05) (Figure 6(a)). Compared with group N, the total histone H4 acetylation level was enhancing in group CIA; however, there was no significant difference between the two groups. Compared with group CIA, the total histone H4 acetylation level was reduced in group WTD; however, there was no significant difference between the two groups (Figure 6(b)).

## 4. Discussion

Based on the character of herbs, WTD has been wildly employed in the treatment of RA. However, the mechanism by which WTD acts on RA is unclear. Systems biology-based investigation indicated that the predicted effect or molecules of WTD were significantly enhanced in neuroactive ligand-receptor interaction and calcium signaling pathway [9]. As the primary component of WTD, the methanol extracts of* Aconitum* roots have shown inhibition of hind paw edema produced by carrageenin in mice [21]. Seventy-four components of WTD have been identified by applying an ultra performance liquid chromatography coupled with quadrupole time-of-flight mass spectrometry method [22]. The study focuses on investigating the potential epigenetic mechanisms of WTD in rats with CIA.

CIA is a typical experimental autoimmune disease that is widely used as a model of RA. CIA model was firstly established by Trentham and colleagues [23]. There are substantial differences in histopathological characteristics of synovium even in two knees of a rat with CIA. Consequently, it is difficult to compare the pathological difference among the groups. For the reasons outlined above we only elucidated the histopathological characteristics of synovial membrane corresponding to AI score.

DNA methylation, the most characterized epigenetic mark, occurs by the covalent addition of a methyl group at the 5-carbon of the cytosine ring by a family of DNMTs with S-adenosyl-methionine as the methyl source, resulting in 5-methylcytosine. DNA methylation is catalyzed by DNMTs, predominantly including DNMT1, DNMT3a, and DNMT3b. DNMT1 is the most abundant DNMT in mammalian cells and is considered to be the key maintenance DNMT in mammals. DNMT1 principally methylates hemimethylated CpG dinucleotides in the mammalian genome. DNA methylation suppresses the expression of genes at transcriptional level [24].

Our results were consistent with the findings of Liu et al. concerning the higher expression of DNMT1 mRNA in group CIA than that in N [25]. Compared with group CIA, the DNMT1 mRNA expression was significantly lowered in group WTD. The global DNA methylation level was significantly reduced in group WTD compared to that in group CIA, which may result from the lower expression of DNMT1 mRNA in group WTD. However, tumor necrosis factor *α* (TNF*α*) blocker has no impact on DNMT1 mRNA expression in RA patients [25]. The different mechanisms of WTD and TNF*α* blocker in the treatment of RA may contribute to the differences.

Human MBDs are a family of methyl CpG binding domain proteins consisted of methyl CpG binding protein 2 (MECP2), MBD1, MBD2, MBD3, and MBD4. Apart from MBD3, each of these proteins is capable of binding specifically to methylated DNA. MECP2, MBD1, and MBD2 can also bind to histone deacetylases (HDACs) functioning as transcription repressors [24]. MBD2 selectively binds to methylated DNA and may function as a mediator of the biological consequences of the methylation signal [26].

The MBD2 mRNA expression was increasing in group CIA compared to that in N; however, there was no significant difference between the two groups. Clinical study also demonstrated that the MBD2 mRNA expression was higher in RA patients [25]. However, the MBD2 mRNA expression was decreasing in group WTD compared with group CIA, although there was no significant difference between the two groups. Anti-TNF*α* biological agents do not seem to affect mRNA expression of MBD2 in RA patients [25]. These suggest that anti-TNF*α* biologics and WTD could not influence MBD2 mRNA expression in treating RA.

Current researches also showed that DNA hypomethylation exists in synovial fibroblasts, T cell, and PBMCs in RA patients [25, 27, 28], indicating that DNA hypomethylation is associated with the pathogenesis of RA. MBD2 participates in silencing of methylated genes [29] and MBD2 activates gene demethylation [30]. It was assumed that group CIA had a higher expression of DNMT1 mRNA with the succeeding global hypermethylation of DNA and then led to a higher expression of MBD2 mRNA through a feedback mechanism.

Histone methylation and histone acetylation, two posttranslational modifications of histones, are investigated intensively for their critical roles in modulating gene transcription. Histone methylation marks can be correlated with transcriptional activation or silencing, dependent on the position and the degree of methylation [31]. The trimethyl mark on H3-K4 (H3-K4me3) was often associated with active transcription [32]. Enhancer of zeste homologue 2 (EZH2) generated the trimethyl mark on H3-K27 (H3-K27me3) which correlated with gene silencing [32, 33].

Our experiments suggested that the mono-, di-, and trimethylated H3-K4 and H3-K27 had no significant increase in group WTD compared to that in group CIA. EZH2, a histone methyltransferase enhancer, was overexpressed in RA synovial fibroblasts (SF) compared with osteoarthritis (OA) SF [34]. However, the studies regarding histones H3-K4 and H3-K27 methylation are rare. Further studies are needed to clarify the role of histone methylation in RA.

Histone acetylation is regulated by the opposite activity of two enzyme families, histone acetyl transferases (HATs) and HDACs. Histone acetylation is catalyzed by HATs, enhancing the rate of gene transcription. On the other hand, histone deacetylation is catalyzed by HDACs, leading to transcriptional silence of certain genes. The imbalance between histone acetylation and deacetylation regulates the transcription rates of various genes and has been indicated to be related to disease states [35].

Our data indicated that the total histone H3 acetylation in group WTD was significantly elevated compared to that in group CIA. HDAC1 was overexpressed in RASF compared to OASF, supporting cell proliferation and survival of RASF [36]. Meanwhile, HDAC2 also played a crucial role in cell proliferation and apoptosis of RASF [36]. In addition, nuclear HDAC activity and expression of HDAC1 were significantly elevated in RA compared to those in OA synovial tissues [37]. A wide range of HDAC inhibitors (HDACis) showed protective effects in prophylactic and therapeutic models of RA [38]. Trichostatin A (TSA), a nonselective HDACi, could potently inhibit the lipopolysaccharide (LPS)-induced production of TNF and interleukin-6 (IL6) in both RA and healthy PBMCs [39]. The HDAC-3-selective inhibitor MI192 inhibited TNF production at high concentrations and dose-dependently reduced IL6 in RA PBMCs but not healthy PBMCs [39]. Class I/II HDACi TSA as well as class III HDAC nicotinamide blocked the TNF*α*-stimulated expression of IL6 and the LPS-induced expression of IL6 and TNF*α* in macrophages of RA patients [38]. TSA time-dependently increased the acetylation of H3 and H4 in macrophages [38]. However, incubation of macrophages with nicotinamide failed to induce detectable acetylation of H3 or H4 [38]. WTD may function as a nonselective HDACi, resulting in the acetylation of H3.

## 5. Conclusions

In recent years, DNA methylation and histone modifications were involved in the pathogenesis of RA. Moreover, numerous epigenetics-based drugs were employed in attenuating the inflammatory activity of RASF or macrophages, especially various selective or nonselective HDACis. WTD may serve as a traditional drug in alleviating disease activity of RA via DNA methylation and histone modifications. The methylated loci and other epigenetic changes of RA are needed to be investigated to confirm this.

## Figures and Tables

**Table 1:** The composition of herbal formula WTD.

Crude herbs	Content	Main components
Ephedra (*Herbal Ephedrae*)	9	Ephedrine, D-pseudoephedrine
Red Peony Root (*Radix Paeoniae Rubra*)	4.5	Paeoniflorin
White Peony Root (*Radix Paeoniae Alba*)	4.5	Paeoniflorin
Root of Membranous Milkvetch (*Radix Astragali*)	9	Astragaloside
Prepared Liquorice Root (*Radix Glycyrrhizae Preparata*)	9	Glycyrrhizic acid
Prepared Monkshood Mother Root (*Radix Aconiti Preparata*)	6	Aconitine, mesaconitine

WTD: Wutou decoction.

**Table 2:** Primer sequence.

Gene	Primer	Sequence
*β*-actin	Forward	5′-CGTTGACATCCGTAAAGACCTC-3′
	Reverse	5′-TAGGAGCCAGGGCAGTAATCT-3′

DNMT1	Forward	5′-CGCTCATTGGCTTTTCTACCG-3′
	Reverse	5′-AGAACTCGACCACAATCTT-3′

MBD2	Forward	5′-AATGATGAGACCCTTCTGTCTGCC-3′
	Reverse	5′-TCCTCTAGTTTCTTTCGGACTTGTTG-3′

DNMT1: DNA methyltransferase 1; MBD2: methyl CpG binding domain 2.
